# Substantial expression of luteinizing hormone-releasing hormone (LHRH) receptor type I in human uveal melanoma

**DOI:** 10.18632/oncotarget.1379

**Published:** 2013-09-10

**Authors:** Andrea Treszl, Zita Steiber, Andrew V. Schally, Norman L Block, Balazs Dezso, Gabor Olah, Bernadett Rozsa, Klara Fodor, Armin Buglyo, Janos Gardi, Andras Berta, Gabor Halmos

**Affiliations:** ^1^ Department of Biopharmacy, University of Debrecen, Medical and Health Science Center, Debrecen, Hungary; ^2^ Department of Ophthalmology, University of Debrecen, Medical and Health Science Center, Debrecen, Hungary; ^3^ Veterans Affairs Medical Center Miami, FL, South Florida VA Foundation for Research and Education, Miami, FL and Department of Pathology, University of Miami, Miller School of Medicine, Miami, FL, USA; ^4^ Divisions of Hematology/Oncology and Endocrinology, Department of Medicine, University of Miami, Miller School of Medicine, Miami, FL, USA; ^5^ Department of Pathology, University of Debrecen, Medical and Health Science Center, Debrecen, Hungary; ^6^ Department of Endocrinology, University of Szeged, Faculty of Medicine, Szeged, Hungary

## Abstract

Uveal melanoma is the most common primary intraocular malignancy in adults, with a very high mortality rate due to frequent liver metastases. Consequently, the therapy of uveal melanoma remains a major clinical challenge and new treatment approaches are needed. For improving diagnosis and designing a rational and effective therapy, it is essential to elucidate molecular characteristics of this malignancy. The aim of this study therefore was to evaluate as a potential therapeutic target the expression of luteinizing hormone-releasing hormone (LHRH) receptor in human uveal melanoma. The expression of LHRH ligand and LHRH receptor transcript forms was studied in 39 human uveal melanoma specimens by RT-PCR using gene specific primers. The binding charachteristics of receptors for LHRH on 10 samples were determined by ligand competition assays. The presence of LHRH receptor protein was further evaluated by immunohistochemistry. The expression of mRNA for type I LHRH receptor was detected in 18 of 39 (46%) of tissue specimens. mRNA for LHRH-I ligand could be detected in 27 of 39 (69%) of the samples. Seven of 10 samples investigated showed high affinity LHRH-I receptors. The specific presence of full length LHRH receptor protein was further confirmed by immunohistochemistry. A high percentage of uveal melanomas express mRNA and protein for type-I LHRH receptors. Our results support the merit of further investigation of LHRH receptors in human ophthalmological tumors. Since diverse analogs of LHRH are in clinical trials or are already used for the treatment of various cancers, these analogs could be considered for the LHRH receptor-based treatment of uveal melanoma.

## INTRODUCTION

Although uveal melanoma is very rare, it is the most common primary intraocular malignancy in adults. Its incidence in the Western world seems to be relatively stable with about 7 new cases per year per 1 million individual. About half of the patients already have metastatic disease by the time of diagnosis. The outcome of these is almost invariably fatal and death usually occurs within a year of the onset of systemic symptoms. [1,2] Treatment by systemic or intra-hepatic chemotherapy or partial hepatectomy only rarely prolongs the survival [3], emphasizing the need to develop more efficacious therapies. Recent progress in our understanding of the molecular processes underlying uveal melanoma should enable us to advance the diagnosis, prognosis and treatment of this malignancy.

The discovery of specific receptors for peptide hormones on cancer cells has led to the development of cytotoxic and radiolabeled hormone analogs that are useful for tumor localization and targeted therapy. Various preclinical studies have shown that chemotherapy based on cytotoxic peptide conjugates targeted to receptors on tumors can improve the effectiveness of treatment and reduce general side effects. [4]

The presence of different isoforms of Luteinizing Hormone-Releasing Hormone (LHRH) also known as Gonadotropin Hormone-Releasing Hormone has been identified in vertebrates. LHRH is the primary link between the brain and the pituitary in the regulation of gonadal function and plays a pivotal role in vertebrate reproduction. The actions of LHRH are mediated by high affinity receptors for LHRH. The discovery of LHRH has had a major impact in medicine and has led to a variety of clinical uses of LHRH analogs in oncology and gynecology. [5] Recently, it has been shown that various cancer cell lines, including cutaneous melanoma, xenografted into nude mice, can be inhibited by the targeted cytotoxic LHRH analog AN-152 (AEZS-108). [6] Since both cutaneous melanoma and uveal melanoma have the same neuroectodermal origin, but the presence of LHRH receptors has never been studied in uveal melanoma, we investigated the expression of mRNAs for LHRH-I ligand and for type I LHRH receptor in specimens of human uveal melanoma. The presence and binding characteristics of LHRH receptor protein were also examined.

## RESULTS

### Expression of human type-I LHRH receptors in human uveal melanoma

**Figure 1:**
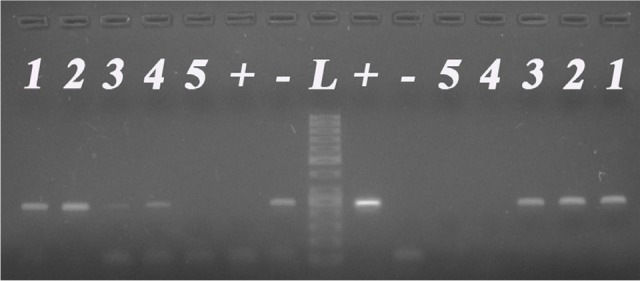
mRNA expression of LHRH-I (left panel) and its type I receptor (right panel) in the same tissue specimen set L: 50 bp DNA-ladder (Fermentas); +: positive control (human pituitary); -: no template control; No. 1-5: representative human uveal melanoma tissues.

**Figure 2:**
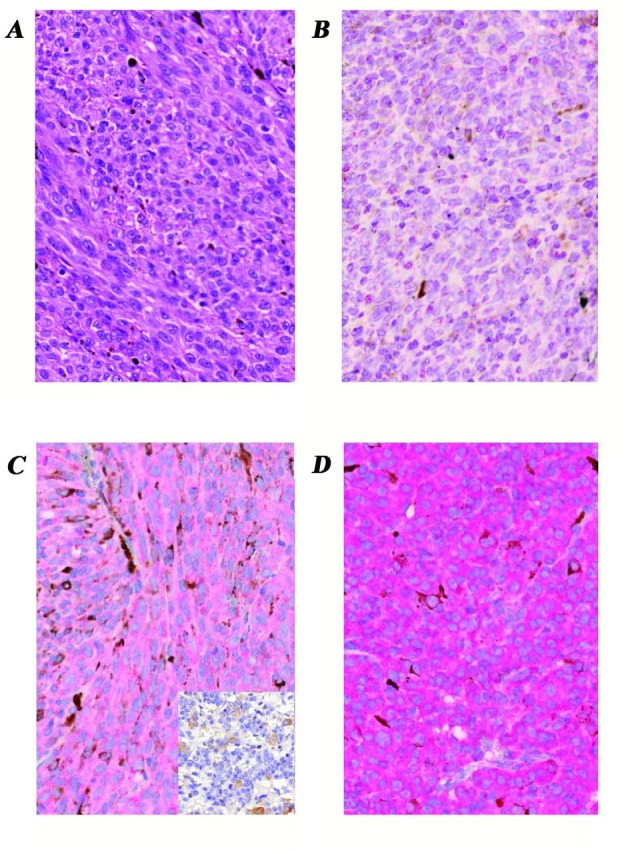
Expression of LHRH receptor protein in enucleated human uveal melanoma tissue samples demonstrated by immunoperoxidase staining A, Hematoxylin-eosin stained section of a representative sample shows melanin-producing neoplastic cells with spindle and epithelioid pattern. a-d, images of representative samples immunostained for type I LHRH receptor; B, tumor sample without detectable mRNA for type I LHRH receptor also revealed no identifiable LHRH receptor protein using IHC staining; C, representative tissue section shows mild positivity for type I LHRH receptor (faint red cytoplasms of tumor cells). Insert is a negative control for the staining-specificity (see Methods); D, representative tumor sample exhibits intense expression of type I LHRH receptor in nearly 100% of tumor cells (intense red cytoplasmic staining) which correlated with the corresponding mRNA levels. Original magnifications of all images: 40x. Images B-D are immunoperoxidase stained sections with hematoxylin nuclear counterstaining.

Our specimens of uveal melanoma tissue consisted of 11 epithelioid, 21 spindle and 7 mixed cell type tumors. The tumor thickness range was 6-12.9 mm according to ultrasonography. The tumor basal diameters as measured with ultrasonography, ranged from 9-19 mm. If the tumor thickness was more than 8 mm and/or the largest tumor diameter was more than 13 mm, we enucleated the eye without prior treatment. In those cases where the thickness was less than 8 mm or the basal diameter was less than 13 mm, but the tumor was growing in spite of the previous transpupillary thermotherapy and/or Ruthenium-106 plaque brachytherapy, enucleation was performed. Based on our recent knowledge, type I LHRH receptor has two splice variants, but only the full length receptor is functional. Our primer set for LHRH receptor was designed to specifically amplify the mRNA of the full length receptor, but to give no signals for the splice variants. We used LHRH receptor primers encompassing the open reading frame from exon 2 to exon 3, overlapping the missing part in the above mentioned two isoforms. The predicted size of the PCR amplified cDNA for type I LHRH receptor was 241 bp. Fourty six percent of our samples expressed receptors for type I LHRH receptor (Fig. 1., Table 1.). Among epithelioid type uveal melanomas, 6 of 11 (55 %) were found to be positive for the expression of LHRH receptor while spindle type melanomas included 10 of 21 (48%) positive samples. In the mixed cell type group (containing both epithelioid and spindle cells, with no dominant pattern), 2 of 7 (29%) of the tumors expressed type I LHRH receptor. The presence of full length LHRH-I receptor was confirmed by immunohistochemistry and correlated well with the findings by RT-PCR (Fig. 2.). Among the specimens of RT-PCR positive uveal melanoma, the majority stained positive for LHRH receptors which could be detected in the form of red granules.

**Table 1:** Clinicopathological characteristics and RT-PCR results of enucleated uveal melanoma samples

Number of patient	Age at enucleation	Sex	Histology	Earlier therapy	Eye	LHRH-R	LHRH ligand
1.	64	M	Spindle	-	L	+	+
2.	39	M	Epithelioid	Brachytherapy	R	+	−
3.	70	M	Spindle	-	R	−	+
4.	80	F	Epithelioid	-	R	+	−
5.	47	M	Epithelioid	-	L	−	−
6.	65	M	Spindle	-	R	+	−
7.	76	M	Spindle	-	R	+	+
8.	75	F	Spindle	-	L	−	+
9.	84	F	Spindle	Brachytherapy	L	+	+
10.	39	M	Mixed	-	R	−	+
11.	35	M	Spindle	-	L	−	−
12.	30	F	Spindle	-	L	+	+
13.	44	F	Spinde	-	L	−	+
14.	68	F	Spindle	-	L	−	+
15.	76	M	Spindle	-	R	+	+
16.	79	F	Mixed	-	L	−	+
17.	79	F	Epithelioid	-	R	+	+
18.	67	M	Epithelioid	Brachytherapy, TTT	L	+	+
19.	60	M	Epithelioid	Brachytherapy (3x)	R	+	+
20.	51	M	Spindle	-	L	−	+
21.	66	M	Epithelioid	-	R	+	+
22.	72	F	Epithelioid	-	L	−	+
23.	76	M	Spindle	-	L	+	+
24.	55	M	Spindle	-	L	−	+
25.	54	M	Epithelioid	-	R	−	+
26.	75	F	Spindle	-	L	+	+
27.	64	F	Epithelioid	-	R	−	+
28.	61	M	Spindle	-	L	−	+
29.	50	F	Spindle	-	R	+	−
30.	76	M	Mixed	-	L	+	+
31.	38	M	Spindle	-	R	−	+
32.	79	F	Mixed	-	R	−	−
33.	53	M	Epithelioid	-	R	−	+
34.	52	M	Mixed	-	L	−	+
35.	45	M	Mixed	-	R	+	−
36.	70	M	Spindle	-	R	+	−
37.	43	M	Spindle	-	L	−	−
38.	53	M	Mixed	-	L	−	−
39.	51	M	Spindle	Brachytherapy (3x)	L	−	−

L: left, R: right, TTT: Transpupillary Thermotherapy

The presence of specific LHRH binding sites and characteristics of binding of [^125^I][D-Trp^6^]LHRH to membrane receptors on human uveal melanoma tissue was determined using ligand competition assays. Of the 10 specimens examined, 7 showed LHRH receptor binding (Table 2.). Analyses of the typical displacement of radiolabeled [D-Trp^6^]LHRH by the same unlabeled peptide revealed that the one-site model provided the best fit, indicating the presence of one class of high-affinity LHRH receptors in crude membranes derived from human uveal melanoma specimens. The computerized nonlinear curve-fitting and the Scatchard plot analyses of the binding data in the 7 receptor-positive tumor specimens indicated that the single class of binding sites had a mean dissociation constant (Kd) of 3.69 nM (range, 1.35 to 6.36 nM) with a mean maximal binding capacity (Bmax) of 384.5 fmol/mg of membrane protein (range, 251.5 to 511.6 fmol/mg protein). Biochemical parameters essential to establish the identity of specific binding sites were also determined. Thus the binding of [^125^I][D-Trp^6^] LHRH was found to be reversible, time- and temperature-dependent, and linear with protein concentration in the human uveal melanoma specimens examined (data not shown). The specificity of LHRH binding was demonstrated by competitive binding experiments using several peptides structurally related or unrelated to LHRH (data not shown).

The expression of mRNA for LHRH receptors was accompanied by ligand binding in all samples examined. Three of 10 tumor specimens did not exhibit mRNA expression for type I LHRH receptors, or show ligand binding; conversely, all receptor-positive specimens expressed a detectable amount of the receptor gene (Table 2.). There was no evident correlation between receptor binding characteristics or mRNA expression and clinical and pathological findings.

**Table 2:** Expression of mRNA and binding characteristics of type I receptors for LHRH (LHRH-R) in 10 human uveal melanoma specimens

Patient No.	LHRH-R mRNA	Bmax (fmol/mg protein)	Kd (nM)
4.	+	294.6	1.35
6.	+	398.7	1.37
9.	+	264.7	3.73
11.	−	-	-
12.	+	495.7	6.03
15.	+	251.5	4.81
20.	−	-	-
24.	−	-	-
30.	+	511.6	6.36
35.	+	474.7	2.18

### Expression of mRNA for human type I LHRH in human uveal melanoma

In addition to the expression of type-I LHRH receptor, we also studied the presence of LHRH ligand in our uveal melanoma samples. The majority of uveal melanomas (27 of 39, 69%) showed marked expression of mRNA for LHRH-I ligand (Table 1.). The expected size of the PCR product of 245 bp could be detected in 8 of 11 (73%) of epithelioid and 15 of 21 (71%) of spindle type tumors. In the mixed tumor type, 4 of 7 (57%) expressed LHRH-I (Fig. 1.). In 12 tumors (31%), both LHRH-I ligand and receptor were expressed. In 6 samples, only type I LHRH receptors were present, while in 15 melanomas only LHRH-I ligand was present (Tabel 1.).

Statistical analysis showed an association between age and LHRH receptor mRNA and ligand co-expression (p=0.0407): a 10-year increase of age meant an estimated 87% increase in the odds of co-expression (OR=1.867, 95%CI 1.027 to 3.393). Otherwise, there were no correlations between LHRH-I ligand or receptor mRNA expression and tumor subtype or clinical parameters. The clinicopathological characteristics of patients with uveal melanoma and the result of mRNA expression analysis are summarized in Table 1.

## DISCUSSION

Patients with uveal melanoma face a dismal prognosis as eventually about 45% of them die of metastasis, regardless of the fact that the tumor is most often diagnosed and locally cured before any signs of clinical disseminated disease appear. [7] This gave rise to the theory that micrometastases are already present early in the disease process, but remain dormant for years before a clinically detectable macrometastasis develops. [8] Most small and medium-sized uveal melanomas are currently managed by different forms of radiotherapy and local resection. However, it remains unclear what effect this treatment has on patient survival. Systemic treatment options for uveal melanoma are very limited. Adjuvant systemic therapy is mainly used in patients at high-risk of metastasis or in patients who have already developed metastasis, but the response rates to classical chemotherapeutic agents remain as low as 7%–25%. [9] Despite the improvements in diagnosis and therapy of primary uveal melanoma in the last 20–30 years, there has been no significant decrease in metastasis-related deaths. [10,11] Thus, the development of new therapeutic modalities is mandated.

The presence of receptors for LHRH in various cancers and cancer cell lines originating from organs other than those of the reproductive system has been demonstrated by several authors. [6,12-15] It was suggested that the signal-transduction mechanisms mediated by type I of LHRH receptor are different in the pituitary and in cancer cells. [16,17] It appears that in cancer cells LHRH analogs interfere with mitogenic signal transduction of growth-factor receptors and related oncogene products associated with activities of tyrosine kinases. [17,18] It has been shown that cutaneous melanomas also express receptors for LHRH. [6,13] The treatment of melanoma cells by agonists of LHRH or cytotoxic analogs of LHRH significantly inhibits cell proliferation. [6,13] As uveal melanoma and melanoma of the skin are both of neural crest origin and share certain genetic characteristics, we analyzed the expression of LHRH receptors in uveal melanoma by RT-PCR, ligand competition assay and immunohistochemistry. As only the full length type I LHRH receptor is functional, our primer set was designed to selectively amplify receptor mRNA encoding the full length type I LHRH receptor but not its known splice variants. The antibody used for immunohistochemistry was also chosen to detect full length protein. In our present study, we found that 47% of our samples expressed type I receptors for LHRH. Furthermore, using ligand competition assay we examined the binding of [^125^I][D-Trp^6^]LHRH to membrane preparations of 10 uveal melanoma specimens. We found that 70% of the human uveal melanoma samples investigated possessed specific type I LHRH receptors with a mean Kd of 3.69 nM and with a mean Bmax of 384.5 fmol/mg membrane protein. It is also important to note that all receptor-positive specimens expressed a detectable amount of the receptor gene. The receptor protein encoded by mRNA for type I LHRH receptors was also demonstrated by immunohistochemistry in tumor specimens.

The high incidence of positivity for type I LHRH receptor in uveal melanoma suggests that this tumor type might be a good candidate for therapy with LHRH analogs including the targeted cytotoxic peptide, AN-152 (AEZS-108). AN-152 is already in phase III clinical trials in women with endometrial and ovarian cancers [18-20] and in phase I/II trials in men with castration resistant prostate cancer [21], Liu S, Schally AV, Dorff TB, Tsao-Wei DD, Groshen SG, Xiong S, Hawes D, Quinn DI, Tai YC, Block NL, Engel J, Pinski JK. A phase I/II trial of AN->152, a targeted cytotoxic LHRH analog, in castration- and taxane-resistant prostate cancer. ASCO Annual Meeting June 3-7, 2011, Chicago, Abstract #74003] and patients with urothelial carcinoma [Fernandez GL, Schally AV, Koru-Sengul T, Merchan JR, Flores AM, Jorda M, Datar R, Benedetto PW, Singal R, Block NL, Engel J. A phase I/II trial of AEZS-108 in locally advanced unresectable or metastatic luteinizing hormone-releasing hormone (LHRH) positive urothelial carcinoma (UC) patients who failed platinum based chemotherapy. ASCO Annual Meeting June 3-7, 2011 Chicago, Abstract # 83230]. Targeted therapy with cytotoxic peptide analogs consisting of a peptide molecule conjugated to a cytotoxic moiety such as doxorubicin, should be more effective and less toxic than conventional systemic chemotherapy [20,22]. Their only side effect appears to be myelosuppression caused by the occasional chemical cleavage of the cytotoxic radical doxorubicin. [4,5,18-20,22] The substantial expression of LHRH-I ligand (69%) and frequent co-expression of its receptor (31%) in our sample set may be indicative of the presence of an autocrine/paracrine regulatory system based on LHRH in uveal melanoma. The regulation of several proteins associated with cell proliferation and cell motility is mediated by type I LHRH receptor/LHRH-I system, suggesting their important role in metastasis formation. [23] However, the role of this high expression of LHRH found by us is not clear.

To the best of our knowledge, our findings represent the first identification of LHRH and its receptors in human uveal melanoma. Since the therapy for this malignancy is not adequate, our work may help to identify specific molecular targets for the prevention of metastasis or further proliferation of already disseminated metastases. Our findings that a high percentage of human uveal melanoma specimens express receptors for LHRH support the view that targeted cytotoxic LHRH analogs such as AN-152 could be used for an effective treatment of uveal melanoma.

## METHODS

### Patients and Tissue Samples

Human uveal melanoma specimens were obtained from 39 patients, 30-84 years of age at the time of enucleation, at the Department of Ophthalmology, University of Debrecen, Hungary. Normal pituitary samples (anterior lobe) were collected at autopsy at the Department of Pathology, University of Debrecen and were used as positive controls. After surgical removal, selected portions of the melanoma tissues were flash frozen and stored at -70°C. Histopathological examination of each specimen was undertaken to confirm the diagnosis. The local Institutional Ethics Committee approved the collection and use of these specimens for the current study and informed consent was obtained from these patients.

### RNA isolation, Reverse transcription and RT-PCR

Total RNA was isolated using AllPrep DNA/RNA/Protein Mini kit according to the manufacturer's instructions (Qiagen, Hilden, Germany). Two hundred fifty nanograms of RNA from each sample were reverse transcribed into cDNA by QuantiTect Reverse Transcription kit (Qiagen) in a final volume of 20 μl. Two primer sets were designed to evaluate the expression of type I LHRH receptors (sense: 5'-GGTGGCATCAAGCATTTTAT-3', antisense: 5'–ACATAGTAGGGAGTCCAGCAGACA-3') and LHRH ligand (sense: 5'–GGCCTTATTCTACTGACTTGG-3', antisense: 5'-TCTTCTGCCCAGTTTCCTCT-3'). As internal control, β-actin housekeeping gene (sense: 5'-GGCATCCTCACCCTGAAGTA-3', antisense 5'-GGGGTGTTGAAGGTCTCAAA-3') was used. In all PCR reactions, 1 μl of cDNA was amplified in a 25 μl solution containing 1.5 mM MgCl2, 1x PCR buffer (Fermentas GmbH, St. Leon-Rot, Germany), 0.3 mM of each deoxynucleotide (Promega, Madison, WI), 1 unit of TrueStart HotStart DNA polymerase (Fermentas) and 0.25 μM of each primer. Samples were denatured for 3 min at 95°C, then subjected to 40 cycles at 95°C for 45 s, 59°C for 30 s, then 72°C for 1.5 min with a final extension at 72°C for 10 min. Ten μl of each amplification reaction was then electrophoretically separated on 1.5% agarose gel, stained with ethidium bromide, and visualized under UV light.

### Preparation of membranes and radioligand binding studies

Preparation of membranes for receptor studies was performed as described previously. [24] Briefly, the samples were thawed, cleaned, and then homogenized in 50 mM Tris-HCl buffer (pH 7.4), supplemented with protease inhibitors (0.25mM Phenylmethylsulfonyl Fluoride, 0.4% (v/v) Aprotinin and 2 μg/ml Pepstatin A) using an Ultra-Turrax tissue homogenizer (IKA Works, Wilmington, NC) on ice. The homogenate was centrifuged at 500x g for 10 minutes at 4 °C to remove nuclear debris and lipid layer. The supernatant containing the crude membrane fraction was ultracentrifuged (Beckman L8-80 M) twice at 70,000x g for 50 minutes at 4 °C after resuspending in fresh buffer. The final pellet was resuspended in homogenization buffer and stored at -80 °C until assayed. Protein concentration was determined by the method of Bradford using a Bio-Rad protein assay kit (Bio-Rad Laboratories, Hercules, CA).

Radio-iodinated derivatives of [D-Trp^6^]LHRH were prepared by the chloramine-T method and purified by reverse-phase HPLC in our laboratory. [24] LHRH receptor binding assays were carried out as reported [24] using *in vitro* ligand competition assays based on binding of [^125^I][D-Trp^6^]LHRH as radioligand to uveal melanoma membrane fractions. This radioligand has been well-characterized previously and shows high-affinity binding to human and rat pituitaries as well as human breast, prostate, and other cancers. [4-6,22,24] In brief, membrane homogenates containing 50-160 μg protein were incubated in duplicate or triplicate with 60-80,000 cpm [^125^I][D-Trp^6^]LHRH and increasing concentrations (10^−12^ - 10^−6^ M) of nonradioactive peptides as competitors in a total volume of 150 μl of binding buffer. At the end of the incubation, 125 μl aliquots of suspension were transferred onto the top of 1 ml of ice-cold binding buffer containing 1.5% bovine serum albumin in siliconized polypropylene microcentrifuge tubes (Sigma-Aldrich GmbH, Munich, Germany). The tubes were then centrifuged at 12,000x g for 3 minutes at 4 °C (Beckman J2-21M). Supernatants were aspirated and the bottoms of the tubes containing the pellet were cut off and counted in a gamma counter (Micromedic System, Huntsville, AL). Preliminary experiments were performed with membrane protein concentrations ranging from 20-250 μg/tube in order to determine the minimal amount of protein required to assess specific binding at a satisfactory level. Our work showed that accurate results can be obtained over a range of 40-180 μg of membrane protein in an incubation volume of 150 μl.

### Immunohistochemistry (IHC)

Formalin-fixed paraffin-embedded tissue samples from enucleation were immunostained as described earlier. [24] Briefly, following antigen-retrieving (pH: 6.0) and endogenous peroxidase-block, 3 μm thick sections were incubated with monoclonal antibody to LHRH-RI (NCL-GnRHR A9E4; Novocastra Laboratories Ltd., UK) at room temperature for 1 hour. After rinsing 3 times in phosphate-buffered saline (PBS; pH:7.4, 5 mins each), sections were treated with anti-mouse IgG (Fab)2-coupled to horse-radish-peroxidase (HRP) of EnVision+-HRP detection kit using amino-ethyl-carbasol (AEC; red; Vector Labs, UK) peroxidase substrate according to the manufacturer's instructions. The use of red chromogenic substrate avoided the color-interference between the brown melanin pigments of melanomas and the positive staining of IHC. Human pituitary glands (anterior lobe) obtained from autopsy were used as positive controls. Negative controls in which primary antibody was replaced by normal serum were also included for each IHC-run.

### Statistical analysis

Variables were described using standard statistics. Association between categorical variables were assessed using Fisher's exact tests, while those between continuous explanatory variables and binary outcomes were assessed using logistic regression, and expressed in terms of odds ratio (OR) and 95% confidence intervals (CI).
